# Quantifying the relationship between sequence and three-dimensional structure conservation in RNA

**DOI:** 10.1186/1471-2105-11-322

**Published:** 2010-06-15

**Authors:** Emidio Capriotti, Marc A Marti-Renom

**Affiliations:** 1 Structural Genomics Unit, Bioinformatics and Genomics Department, Centro de Investigación Príncipe Felipe, Valencia, Spain; 2 Current Address: The Helix Group, Department of Bioengineering, Stanford University, Stanford, CA 94305-5444, USA

## Abstract

**Background:**

In recent years, the number of available RNA structures has rapidly grown reflecting the increased interest on RNA biology. Similarly to the studies carried out two decades ago for proteins, which gave the fundamental grounds for developing comparative protein structure prediction methods, we are now able to quantify the relationship between sequence and structure conservation in RNA.

**Results:**

Here we introduce an all-against-all sequence- and three-dimensional (3D) structure-based comparison of a representative set of RNA structures, which have allowed us to quantitatively confirm that: (i) there is a measurable relationship between sequence and structure conservation that weakens for alignments resulting in below 60% sequence identity, (ii) evolution tends to conserve more RNA structure than sequence, and (iii) there is a twilight zone for RNA homology detection.

**Discussion:**

The computational analysis here presented quantitatively describes the relationship between sequence and structure for RNA molecules and defines a twilight zone region for detecting RNA homology. Our work could represent the theoretical basis and limitations for future developments in comparative RNA 3D structure prediction.

## Background

The view of RNA as a simple information transfer molecule has been challenged since the discovery of ribozymes, a class of RNA with enzyme-like functions [1-3]. RNA molecules are now known to carry a large repertory of biological functions such as transfer of information, enzymatic catalysis and regulation of cellular processes [4]. Similar to proteins, functional RNA molecules fold into specific three-dimensional conformations essential for performing their biological activity. Despite advances in characterizing the folding and unfolding of RNA molecules [5-8] and the significant increase of RNA structures deposited in the Protein Data Bank (PDB) [9], our knowledge of the atomic mechanism by which RNA molecules adopt their biological active structures is still limited [10]. Nonetheless, it is common knowledge that RNA 3D structure is more conserved than RNA sequence and that such principle could be used for comparative RNA structure prediction in a similar way it is done for proteins [11]. It was back in the eighties when Chothia and Lesk first quantified such evolutionary relationship for proteins [12-14]. Their seminal works on the relationship between protein sequence and structure conservation provided the theoretical grounds for many computational approaches in comparative protein structure and function prediction [11,15]. Their work concluded that the overall structural changes between two homologous proteins were proportional to their sequence differences. It was then estimated that homologous proteins aligning with less than 20% sequence identity could have large structural differences [14]. Such findings were later confirmed and expanded by several other studies [16-20].

For RNA, the axiom of "function is more conserved than structure and structure is more conserved than sequence" has been adopted since the end of the sixties [21] and even reinforced with the analysis of newly determined large RNA containing complexes such as the ribosome [22-29]. The wealth of new structures has prompted the development of computational methods for classifying RNA molecules [30-34], describing their structural features [35-37] assigning their functions [34,38] comparing their structures [39-41] and predicting their structures [42-45]. For example, the relationship between sequence and structure in RNA molecules has previously been characterized for the RNA ribose zipper [28], the C1 region of the env HIV1 gene [46] and RNA loop regions [47,48]. A new method that relies on secondary structure information to align homolog RNA sequences was also recently developed [49]. Finally, with the aim of characterizing RNA structure diversity, Abraham and co-workers recently studied the RNA conservation at three levels: primary, secondary, and tertiary structure. The work resulted in the DARTS database [34], which constitutes a new classification of RNA structure after the SCOR database [31]. However, to date no general large-scale study has systematically addressed the quantitative analysis of the relationship between sequence and structure conservation in RNA molecules. The work here introduced aims to address such gap by performing an all-against-all comparison of 451 non-identical (that is, 100% sequence identity) RNA structures from the PDB. The resulting analysis confirms in a general and quantitative manner the relationship between sequence and structure conservation in RNA molecules as well as allows the definition of a "twilight zone" for RNA homology detection.

We begin this article by describing the results of an all-against-all comparison of a non-identical RNA structure set (Results). Next, we discuss the impact that our findings could have in the development of comparative approaches for RNA structure prediction (Discussion). Finally, we end by detailing the methodology used for aligning and assessing RNA alignments (Materials and Methods).

## Results

### Accurate pair-wise alignments of RNA structures

**Figure 1:**
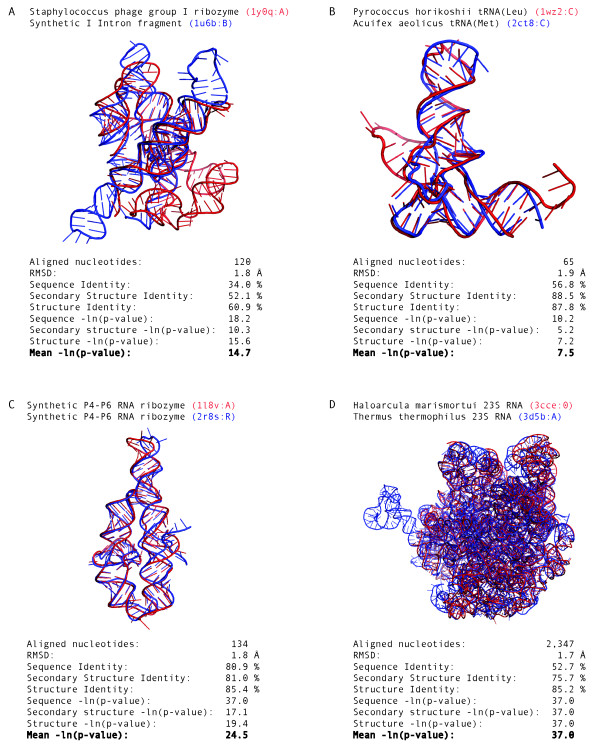
**Accurate RNA structure alignments**. (A) *Staphylococcus phage *group I ribozyme (1y0q PDB identifier, chain A) superimposed to a fragment a synthetic construct group I Intron (1u6b PDB identifier, chain B). (B) tRNA(Leu) of *Pyrococcus horikoshii *(1wz2 PDB identifier, chain C) superimposed to the tRNA(Met) of *Acuifex aeolicus *(2ct8 PDB identifier, chain C). (C) Superimposition of two synthetic constructs P4-P6 RNA ribozyme domain (1l8v and 2r8s PDB identifiers, chains A and R). (D) 23S ribosomal RNA of *Haloarcula marismortui *(3cce PDB identifier, chains 0) superimposed to the 23S ribosomal RNA of *Thermus thermophilus *(3d5b PDB identifier, chain A).

All pair-wise RNA structure alignments were generated by the SARA program [41] using the 114 non-identical RNA structures in the HA-RNA09 dataset. The SARA program was run with default parameters and selecting only based-paired regions of the structures (Methods). HA-RNA09 included 589 pair-wise alignments between 39 tRNA (34.2%), 21 23S Ribosomal RNA (18.4%), 14 riboswitches (12.3%), 11 Ribozymes (9.6%), 10 5S Ribosomal RNA (8.8%), 9 16S Ribosomal RNA (7.9%) an 10 other RNA structures (8,8%). Such alignments superposed with PSI values (*i.e*., percentage of C3' atoms within 4.0 Å RMSD) higher than 60% and only 3 alignments resulted in less than 75% PSI. The average PSI value for the 589 pair-wise alignments was 90.4%, which corresponded to a core RNA size ranging from ~50 to ~2,800 nucleotides. The superimposition of the *Staphylococcus phage *group I ribozyme (1y0q PDB identifier, chain A) and a fragment of the synthetic construct group I Intron (1u6b PDB identifier, chain B) resulted in 60.9% aligned C3' atoms, which corresponded to a structural core of 120 nucleotides (Figure 1A). About 48% of the pair-wise alignments in the HA-RNA09 dataset aligned two tRNA molecules. The alignment of a tRNA(Leu) of *Pyrococcus horikoshii *(1wz2 PDB identifier, chain C) and the tRNA(Met) of *Acuifex aeolicus *(2ct8 PDB identifier, chain C) resulted in 65 core nucleotides within 1.9 Å RMSD (Figure 1B). About 14% of the alignments in the HA-RNA09 dataset superposed a pair RNA structures that did not include either a tRNA or a ribosomal RNA. The alignment of two synthetic constructs P4-P6 RNA ribozyme domains (1l8v and 2r8s PDB identifiers, chains A and R, respectively) resulted in 134 core nucleotides within 1.8 Å RMSD (Figure 1C). Finally, ~38% of the alignments in the HA-RNA09 dataset corresponded to alignments between ribosomal RNA molecules. The alignment of 23S ribosomal RNA of *Haloarcula marismortui *(3cce PDB identifier, chain 0) and 23S ribosomal RNA of *Thermus thermophilus *(3d5b PDB identifier, chain A) resulted in 2,347 core nucleotides within 1.7 Å RMSD (Figure 1D).

### The relationship between sequence and structure conservation in RNA

**Figure 2:**
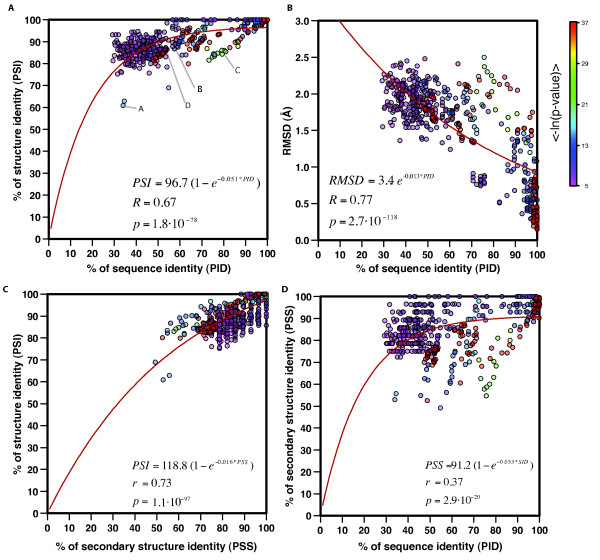
**The relationship between sequence and structure conservation in RNA**. (A) Relationship between sequence and structure conservation in RNA shown for the 589 pair-wise structure alignments in the HA-RNA09 dataset. Points are colored proportional to the mean of the negative logarithm of the three p-value cut-offs (-*ln(P*_*PID*_), -*ln(P*_*PSS*_), and -*ln(P*_*PSI*_)). Example pair-wise alignments are highlighted with the letters of their respective panels in Figure 1. (B) Relationship between RMSD and sequence conservation. (C) Relationship between secondary and tertiary structure conservation. (D) Relationship between sequence and secondary structure conservation.

All the alignments in the HA-RNA09 set superposed a medium to large structurally conserved RNA core. The structure diversity between the selected pairs of RNA structure cores had a measurable relationship to the sequence diversity, which best fitted an exponential decay curve with a 0.67 correlation coefficient (Figure 2A). Similar to proteins, the structure identity decreased with the decrease of the sequence identity and the structure of the core was more conserved than its sequence. The median of the percentage of sequence and structure identity for the 589 alignments in the HA-RNA09 set were 52.3% and 89.0%, respectively. Sequence identity ranged from 29.2 to 100.0%, with 95% of the alignments with PID (*i.e*., percentage of sequence identity) higher than 34.2%. Structure identity ranged from 60.9 to 100.0%, with 95% of the alignments with PSI higher than 81.2%. The same trend is observed by comparing sequence conservation (PID) with structure diversity (structure core RMSD) (Figure 2B). The relationship between RMSD and percentage of sequence identity fits an exponential curve with a 0.77 correlation coefficient. It is important to note that the pair-wise structure alignments appeared to cluster into two groups around sequence identity ranges of 30-60% and 90-100%. The correlation coefficient between RMSD and sequence identity in the two groups was 0.34 (p = 8.7·10^-11^) for 30-60% and 0.63 (p = 3.8·10^-15^) for 90-100%, which indicates that as sequence identity decreases, the relationship between sequence and structure conservation weakens. Furthermore, to assess the impact of the sequence high identity alignments (95-100%) in the fitting of our exponential curves, we removed all pair-wise alignments with sequence identity higher than 95%. The correlation coefficient between RMSD and PID decreased to 0.66 but remained statistically significant (*p *= 1.5·10^-57^). A similar clustering of the sequence space was previously observed for proteins [19,50], where there was an increase of frequency of alignments with low sequence identity (20-40%) as well as high sequence identity (90-100%).

As previously observed, this analysis quantitatively confirmed that RNA secondary structure largely determines tertiary structure. The relationship between secondary structure and tertiary structure conservation in RNA best fitted an exponential decay curve with a correlation coefficient of 0.73 (Figure 2C). The median of secondary structure identity (PSS) for the 589 alignments in the HA-RNA09 set was 85.7%, which agrees with previous analysis [34]. However, there was a weaker relationship between sequence and secondary structure conservation in RNA, which could only be fitted to an exponential decay curve with a correlation coefficient of 0.37 (Figure 2D). Pairs of structures that align with relatively high sequence identity could have different secondary structures. For example, the alignment of two group I introns (1hr2 and 1x8w PDB identifiers, chains A) resulted in 75.6% sequence identity and only 54.7% secondary structure identity.

### A "twilight zone" for RNA sequence alignments

**Figure 3:**
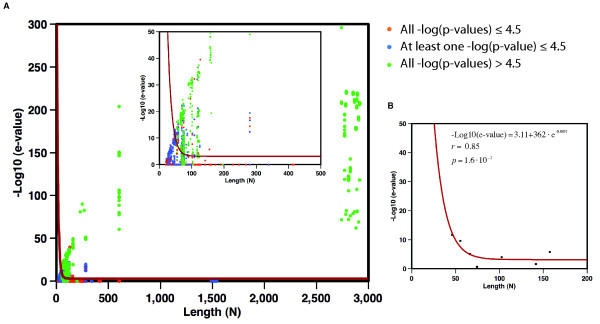
**"Twilight zone" for RNA sequence alignments**. The length of the shorter of the two aligned sequences (*N*) is plotted against the e-value of aligning both sequences with the *Infernal *program. (A) Result from an all-against-all sequence comparison of the RNA sequences in the RNA09 dataset. Green dots correspond to true positive relationships (*i.e*., with -*ln(P*_*PID*_), -*ln(P*_*PSS*_), and -*ln(P*_*PSI*_) higher than 4.5). Blue dots correspond to medium accuracy alignments (*i.e*., with at least one of the three scores below or equal to 4.5). Orange dots correspond to true negatives relationships (*i.e*., with -*ln(P*_*PID*_), -*ln(P*_*PSS*_), and -*ln(P*_*PSI*_) lower or equal to 4.5). The inner plot shows the results for alignments with *N *shorter than 500 nucleotides. (B) The best-fit curve (red line in panels A and B) that optimally separates true positive from true negative. Matthew's correlation coefficient of 0.81 (-Log_10_(e-value) = 3.11+3.62e+02·e^-0.0798N^, fitting correlation coefficient 0.85, probability 1.6·10^-2^).

The 101,475 pair-wise alignments in the RNA09 dataset were divided into three different groups depending on the -*ln(P*_*PID*_), -*ln(P*_*PSS*_), and -*ln(P*_*PSI*_) cut-offs, which were calculated using the background distribution of similarity scores from a set of 3D structural alignments between RNA molecules with sequence identity lower than 25% (Methods). The resulting groups included: (i) true positive alignments between related structures with all three cut-offs higher than 4.5, (ii) true negative alignments between dissimilar structures with all three cut-offs lower than or equal to 4.5, and (iii) medium accuracy alignments with one or two cut-offs higher than 4.5. The sequences of all pair-wise alignments within each group were aligned using the sequence-based alignment program *Infernal *[51]. This division and alignment allowed us to study of relationship between alignment significance (*i.e*., *Infernal *e-value) and the length of the alignment (*i.e*., shortest sequence between the aligned RNA) as well as to assess the difficulty detecting homology based solely on sequence information. Similar to proteins [19], we observed a "twilight zone" for sequence alignment where true relationship was difficult to assess (Figure 3A). The curve that best separated true positive pairs (green dots) from false positive pairs (orange dots) exponentially decayed from 10^-10 ^e-value for RNA sequences of ~50 nucleotides and leveled at ~5·10^-4 ^e-value for RNA larger than 100 nucleotides (Figure 3B). Pair-wise alignments below the "twilight zone" curve included 98,841 true negatives (*i.e*., unrelated pairs below the curve) and only 152 false positives (*i.e*., unrelated pairs above the curve). There were a total of 262 RNA structure pairs with all three p-values higher than or equal to 4.5 that were very difficult to detect based on the *Infernal *alignment score. Those pairs of sequences, which corresponded mainly to tRNA molecules, aligned between 67 and 78 nucleotides with mean sequence identities of 41.7% and constitute a 23.2% of false negative rate (*i.e*., true related pairs below the fitted curve). The analysis of the 262 false negative pair-wise alignments showed that their average PID was much lower (41.7%) than for the pairs of related structure above the "twilight zone" (71.2% average PID). However, the secondary structure of those pairs resulted in an 83.1% average PSS indicating that even though their sequences diverged, their secondary and tertiary structures were conserved. This set thus becomes a very difficult set of related RNA structures to detect by sequence alignment methods.

## Discussion

Our analysis indicate that two related RNA molecules conserve a structure core of at least ~50 nucleotides, which can be superimposed within 4.0 Å RMSD. Such conserved structure core starts diverging as sequence identity decreases below 50% and becomes noteworthy (*i.e*., structural divergence >20%) for pairs of RNA structures that superimpose with sequence identity below 40%. Moreover, the exact relationship between sequence and structure conservation for pairs of distant RNA molecules (that is, resulting in 30 to 60% sequence identity alignments) is less evident, which results in a correlation coefficient of 0.34. Homologous pairs of RNA molecules will diverge into different structures when there is a significant decrease in the identity of their sequences. Therefore, it is more difficult to assess structure conservation based on sequence diversity in the low regime of sequence identity (*i.e*, <60%). Highly similar structures conserve their base pairing. The degree of conservation between tertiary and secondary structure in RNA results in a correlation of 0.73. However, the relationship between sequence and secondary structure conservation is weakly correlated, which agrees with the difficulty of predicting secondary structure from RNA sequence alone. Similar conclusions were obtained with the ARTS program, which used a >90% secondary structure identity threshold for structural classification of RNA [34]. Our results show that for conserved RNA structure cores, high secondary structure identity implies high tertiary structure identity but not necessarily high sequence identity. This reflects the co-variation effect in RNA that requires balancing a single mutation with a second change in the based-paired nucleotide to maintain its secondary and tertiary structures.

To accurately detect a pair of related RNA structures from sequence, their alignment should result in *Infernal *e-values smaller than 5·10^-4^. This result indicate that for RNA, likewise for proteins [19], there is a "twilight zone" for the practical application of homology-based approaches for RNA structure prediction. Using the *Infernal *program with an e-value cut-off of 5·10^-4^, we identified 50,523 pair-wise alignments between RNA sequences from the RFam database [52] and known RNA structures. This represents 26.2% and 4.5% coverage of all sequences and families in the RFam database, respectively. Of those, 90.7% (45,812) were between two sequences that result in alignments above the "twilight zone" curve and represent a set of query sequences for which comparative RNA structure prediction could reliably be used.

It is important to note that our study is currently affected by two circumstances. First, the distribution of RNA structures deposited in the PDB is rather scattered. It is known that the RNA structures in the PDB do not evenly represent the entire RNA structure space. To minimize such problem, we have used a pre-selected dataset of non-identical RNA structures (identity cut-off of 100%) as well as removing alignments between a structure and its sub-structures. Moreover, such problem will become less relevant with time given the increased pace of deposition of new RNA structures in the PDB. Second, RNA motif comparison has classically been centered on small RNA fragments of about 10 to 30 nucleotides. However, given the intrinsic difficulty of discerning significant alignments from random pairs of short RNA structures or motifs, our work has focused on identifying structural cores of at least ~50 nucleotides.

## Conclusions

Despite the increasing interest on RNA function and the accelerated deposition of RNA structures, there was a gap between the common knowledge and a quantitative analysis of the relationship between sequence and structure conservation in RNA. Here we have addressed this knowledge gap by applying our RNA alignment method [41] to a set of 451 non-identical RNA structures. The relationship we quantified confirms previous studies in ribosomal RNA [25,27,28,53] and could prove useful to assess whether a particular alignment could be reliably used for comparative RNA structure prediction. We have quantitatively shown that: (i) there is an exponential decay relationship between sequence and structure conservation, (ii) evolution tends to conserve more structure than sequence, and (iii) there is a "twilight zone" for RNA homology detection.

Our study provides an initial assessment of the current limits of comparative modeling of RNA structures. We anticipate that our work will aid the development of new methods for RNA structure prediction from sequence. In the near future, it is expected that large-scale comparative modeling of RNA structures will extend opportunities for answering key questions about RNA evolution, such as the origin of RNA as functional molecules [54]. We have estimated that it would be possible to model by comparative modeling segments of approximately one quarter of all RNA sequences in the RFam database. More accurate RNA sequence alignment methods, including those that explicitly use base-pairing information, will be needed to increase the coverage, diversity and accuracy of reliable comparative RNA 3D structure models. Assuming the current growth rate in the number of known RNA structures, comparative modeling will be applicable to a significant number of RNA families within the next few years and thus could play an important bridging role in understanding the atomic mechanisms of RNA folding.

## Methods

Two types of RNA alignments were obtained in our experiment. First, 3D structure-based alignments, which were produced by the SARA program [41] and used to characterize the relationship between sequence and secondary/tertiary structure conservation in RNA. Second, sequence-based alignments, which were produced by the *Infernal *program [51] and used to characterize a "twilight zone" for RNA homology detection.

### Structure-based RNA alignments

Pairs of RNA structures were aligned using the SARA program, which is based on the unit vector approach [55]. A similar approach was previously used for protein structure alignment by the MAMMOTH program [56]. Briefly, SARA alignments were built by the following procedure: (i) given a RNA structure with *N *nucleotides, for each *i *= 1,...,*N*-1 extract the vector from the *i*-th to the (*i*+1)-st selected atoms; (ii) normalize all vectors to a unit-distance, and place the tails of all normalized vectors at the origin of a unit-sphere; the resulting collection of normalized vectors is the unit-vector representation of the input RNA. In this work, SARA aligned two RNA structures by selecting only C3' atoms involved in base-pairing as computed by the 3DNA program [57]; (iii) calculate the Unit-vector RMS distance between two sets of unit vectors as a score for local RNA structural comparison. This is equivalent to the root mean square deviation (RMSD) between their corresponding normalized vectors after determining the rotation to minimize the RMSD [58]; (iv) the comparison of consecutive unit-spheres generates an all-against-all similarity scoring matrix, which is used in a dynamic programming procedure for the global alignment of two RNA structures using zero end gap penalties [59]; (v) the output alignment is then refined by maximizing the number of equivalent atoms or base-pairs within 3.5 Å RMSD using a variant of the MaxSub algorithm, which ensures that the best local alignment is contained in the resulting alignment [56,60]; and (vi) three p-value and their negative logarithms are calculated to assess the statistical significance of the resulting alignment scores.

The three alignment scores calculated by SARA are: first, percentage structure identity (PSI):(1)

where *n*_*al *_is the number of aligned C3' atoms within a threshold distance of 4.0 Å and *N *is the length of the shortest of the two RNA structures. Second, percentage of aligned secondary structure (PSS):(2)

where *p*_*al *_is the number of aligned base pairs within a threshold distance of 4.0 Å and *NP *is the smallest number of base pairs of the two aligned RNA structures. Two base pairs are aligned when both C3' atoms of the interacting nucleotides in the first structure are below 4.0 Å to the two C3' of the interacting nucleotides in the second structure. Third, percentage of sequence identity (PID):(3)

where *n*_*id *_is the number of identical nucleotide types aligned in the structural alignment and *N *is the length of the shortest of the two RNA structures.

After an alignment is produced, SARA calculates the PSI, PSS and PID scores as well as their associated p-values for estimating the probability of obtaining an equal- or better-scored alignment by chance. The distribution of the accuracy scores (*i.e*., PSI, PSS and PID) for alignments between unrelated RNA structures follows an extreme value distribution and the probability for a given alignment to obtain a score *x *larger than *z *can be calculated by integrating the Gumbel distribution:(4)

where γ = 0.5772 and *z *is(5)

where μ, σ are the values that best fit the extreme value distribution (Eqn. 4 and Additional file 1 Figure S1).

### Sequence-based RNA alignments

To assess the limits of RNA homology detection, we used the *Infernal *program [51] to generate a set of covariance models (CMs) for each structure in the RNA09 dataset. The CMs were built using known RNA secondary structures calculated by the 3DNA program [57] from the 3D coordinates of the structures. An arbitrary random sequence length of 4 Mb was set to calibrate the local covariance model, which exceed the length of largest ribosomal RNA sequences in the RNA09 dataset (~2900 nt). We then performed a leave-one-out procedure removing from the RNA09 dataset one RNA entry at the time and treating it as target sequence of unknown structure. Each target was aligned with *Infernal *against the remaining set of CMs of known structures or templates. The size of the search space was set to 200 Mb during the search step. All other parameters in *Infernal *were set at their default values. The searching by *Infernal *returned a list of hits of each target sequence and the e-value of the statistical significance of the alignments. *Infernal *resulted in 2,335 top hits with e-value lower than 1.0, which where stored and used to calculate a "twilight zone" for RNA homology detection. Additionally, all the 451 covariance models generated from the RNA structures in RNA09 dataset where used to search homologous RNA sequences in the RFam database using the same *Infernal*-based protocol. Such analysis allowed us to assess the likely impact of comparative RNA structure prediction.

### Alignments datasets

As of March 2009, the PDB database stored a total of 1,534 structure files containing at least one RNA chain. This initial dataset of structures was filtered by removing RNA structures that: (i) were less than 20 nucleotides long and had less than 4 base pairs; and (ii) were 100% identical in sequence to another RNA structure in the dataset. The final dataset (RNA09) included 451 RNA chains from 417 PDB entries (Table 1 and Additional file 2). Next, an all-against-all comparison of the structures in the RNA09 set using the SARA program [41] was carried out and resulted in 101,475 pair-wise RNA structure alignments, which were used to create two other datasets: (i) the non-related structure alignments dataset (NR-RNA09), which included 50,995 pair-wise alignments (Table 1 and Additional file 3) and (ii) the highly accurate structure alignments dataset (HA-RNA09), which included 589 pair-wise alignments (Table 1 and Additional file 4). The NR-RNA09 dataset was generated by selecting only pair-wise alignments in the RNA09 dataset with sequence identity below 25% and was used for calculating the background distribution of identity scores (Additional file 1 Figure S1). The HA-RNA09 dataset was generated by selecting high-scored alignments (*i.e*., with all three negative logarithm of p-value -*ln(P*_*PSI*_), -*ln(P*_*PSS*_), and -*ln(P*_*PID*_) higher than or equal to 4.5) with a crystallographic resolution higher than 5 Å and removing all alignments between a RNA structure and its sub-structures. The ability of the negative logarithm p-values scores for identifying biologically relevant alignments was previously tested by means of functional annotation of the SCOR database [31,38]. The HA-RNA09 dataset, which was generated after applying this triple cut-off to the RNA09 dataset and removing sub-structures, represented a set of *related *non-identical high-resolution RNA structures. Therefore, the HA-RNA09 dataset is a good compromise between the need of a large set of alignments and the appropriate sampling of the sequence and structure spaces. The entire datasets of RNA structures and alignments used in this work are available as Additional files as well as for downloading at http://sgu.bioinfo.cipf.es/datasets.

**Table 1:** RNA structure and alignments datasets.

Dataset	Number of structures	Number of alignments
RNA09	451	101,475
NR-RNA09	451	50,995
HA-RNA09	114	589

## Authors' contributions

EC carried out the computational analysis. EC and MAM-R conceived and designed the study as well as drafted the manuscript. All authors read and approved the final manuscript.

## Supplementary Material

Additional file 1 **P-value parameter optimization**. Fitting of the μ and σ values for the calculation p-values for PID, PSS and PSI. Click here for file

Additional file 2 **RNA09 dataset**. Structural alignments in the NR-RNA09 dataset used in the all-against-all structural comparison. Click here for file

Additional file 3 **NR-RNA09 dataset**. Structural alignments in the NR-RNA09 dataset used for the calculation of the background distributions for PID PSS and PSI. Click here for file

Additional file 4 **HA-RNA09 dataset**. Structural alignments in the HA-RNA09 dataset used to quantify the relationship between sequence and structure in RNA. Click here for file
