# Supplementary material for: Quantifying the relationship between sequence and three-dimensional structure conservation in RNA
Source: BMC Bioinformatics. 2010 Jun 15;11:322. doi: 10.1186/1471-2105-11-322 (PMC2904352; doi:10.1186/1471-2105-11-322)
Supplement: Additional file 1 — P-value parameter optimization. Fitting of the μ and σ values for the calculation p-values for PID, PSS and PSI. [file 1471-2105-11-322-S1.DOC]

**Supporting information to the manuscript:**

**Quantifying the relationship between sequence and three-dimensional structure conservation in RNA.**

Emidio Capriotti and Marc A. Marti-Renom^*^

Structural Genomics Unit. Bioinformatics and Genomics Department.

Centro de Investigación Príncipe Felipe. Valencia. Spain


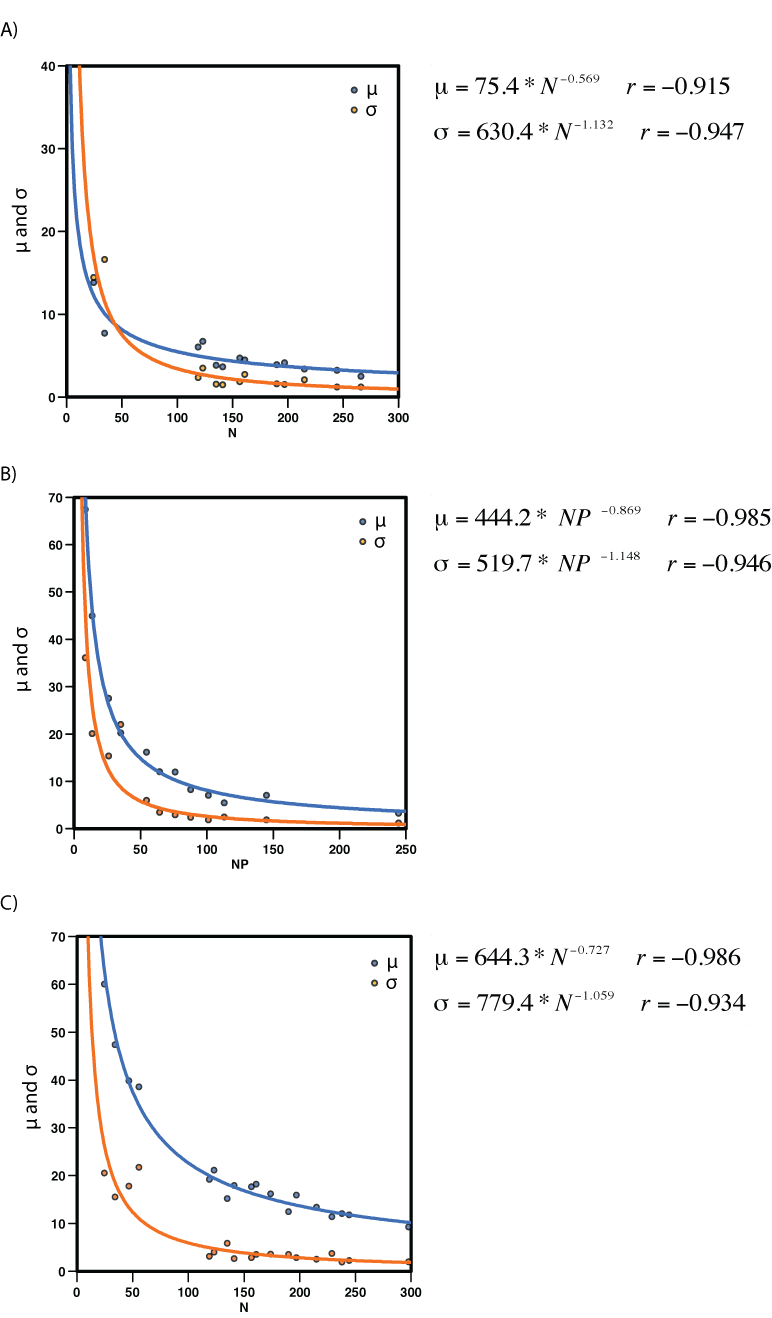


**Figure S1.** Fitting of the μ and σ values. μ (blue) and σ (orange) parameters for PID, PSS and PSI that best fit an extreme value distribution. The distributions have been calculated using a set of 50,995 alignments between pairs of unrelated RNA (*i.e.*, the NR-RNA09 set). The alignments were binned by the length of the shortest RNA (N) for PID and PSI scores and by the lowest number of base pairs (NP) for the PSS score.

**RNA structure datasets:**

| **Dataet** | **Number of structures** | **Number of alignments** | **Dataset file** |
| --- | --- | --- | --- |
| RNA09 | 451 | 101,475 | Additional file 2 |
| NR-RNA09 | 451 | 50,995 | Additional file 3 |
| HA-RNA09 | 114 | 589 | Additional file 4 |
